# Supplementary material for: Colonization of dermal arterioles by Neisseria meningitidis provides a safe haven from neutrophils
Source: Nat Commun. 2021 Jul 27;12:4547. doi: 10.1038/s41467-021-24797-z (PMC8316345; doi:10.1038/s41467-021-24797-z)
Supplement: Supplementary file 1 — Supplementary Information [file 41467_2021_24797_MOESM1_ESM.pdf]

SUPPLEMENTARY INFORMATION

Colonization of dermal arterioles by *Neisseria meningitidis* provides a safe haven from neutrophils

Valeria Manriquez[1], Pierre Nivoit[1], Tomas Urbina[1], Hebert Echenique-Rivera[1], Keira Melican[1, a], Marie-Paule Fernandez-Gerlinger[1], Patricia Flamant[2], Taliah Schmitt[3], Patrick Bruneval[4], Dorian Obino[1]* and Guillaume Duménil[1]*

[1] Pathogenesis of Vascular Infections unit, INSERM, Institut Pasteur, 75015 Paris, France
[2] Experimental Neuropathology Unit, Institut Pasteur, 75015 Paris, France
[3] Paris Saint-Joseph Hospital, 75015 Paris, France
[4] Service d'Anatomie Pathologie, Hôpital Européen Georges Pompidou, Assistance Publique-Hôpitaux de Paris (AP-HP), Paris, France

[a] Current address: Department of Neuroscience, Karolinska Institutet, SE-17177 Stockholm, Sweden

* Correspondence: G.D. (email: guillaume.dumenil@pasteur.fr) and D.O. (email: dorian.obino@pasteur.fr)

Supplementary Figures 1 to 5

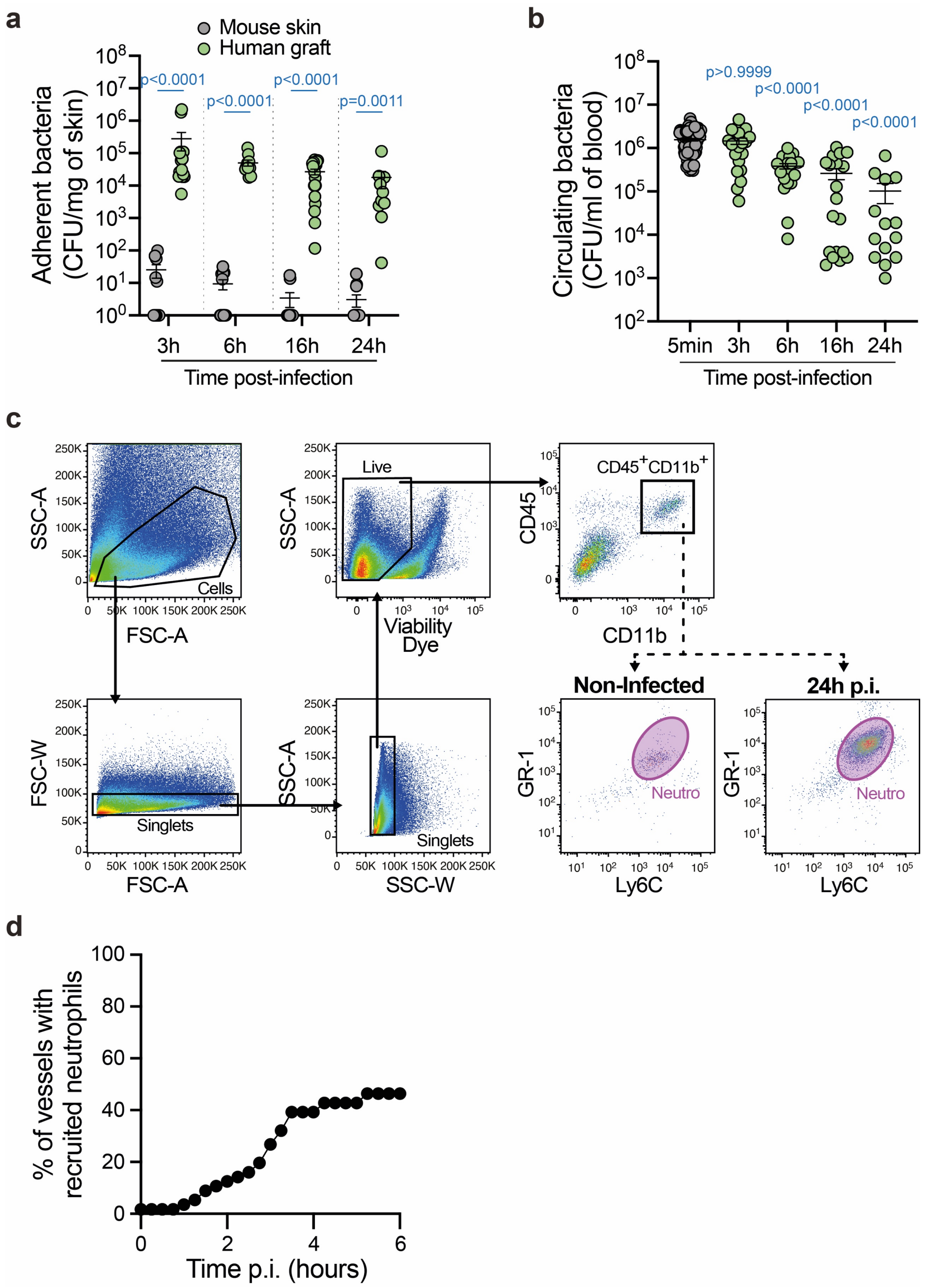

Supplementary Figure 1. Neutrophil recruitment upon vascular colonization. a-b, Bacterial colony forming unit (CFU) counts from **(a)** dissociated skin biopsies (adherent bacteria) collected from either the human graft (green circles) or contralateral mouse skin (grey circles) and **(b)** blood (circulating bacteria) of infected mice at the indicated times post-infection. Data are shown as the mean±SEM. Two-tailed Kruskal-Wallis test with Dunn's correction. For adherent bacteria in **(a)**: n=10 mice (mouse skin, 3h p.i.), 17 mice (xenograft, 3h p.i.), 12 mice (mouse skin 6h p.i. and 16h p.i. and xenograft 6h p.i.), 18 mice (xenograft, 16h p.i.), 15 mice (mouse skin, 24h p.i.) and 11 mice (xenograft, 24h p.i.), pooled from N=5 independent experiments per time point (except for 6h p.i. N=4). For circulating bacteria in **(b)**: n=73 mice for 5 min p.i., 22 mice for 3h, and 6h p.i., 20 for 16h p.i. and 14 for 24h p.i., pooled from N=4 independent experiment per time point (except for 16h p.i. N=5 and 24h p.i. N=3). ns, not significant. **c,** Flow cytometry gating strategy used to quantify murine neutrophils (Neutro) in dissociated mouse skin, human xenografts and blood based on the cell surface expression of CD45, CD11b, Ly-6C, and GR-1. This strategy has been used in Fig. 1c, 2d, 2h, 3b, 3d, 4c, as well as in Supplementary Fig. 3a and 3c. **d,** Movies obtained from intravital imaging were used to quantify the percentage of vessels effectively recruiting neutrophils during the first 6h of the infection. Data are shown as percentage of vessels. Quantifications were performed on n=56 vessels, pooled from N=7 infected mice imaged independently.

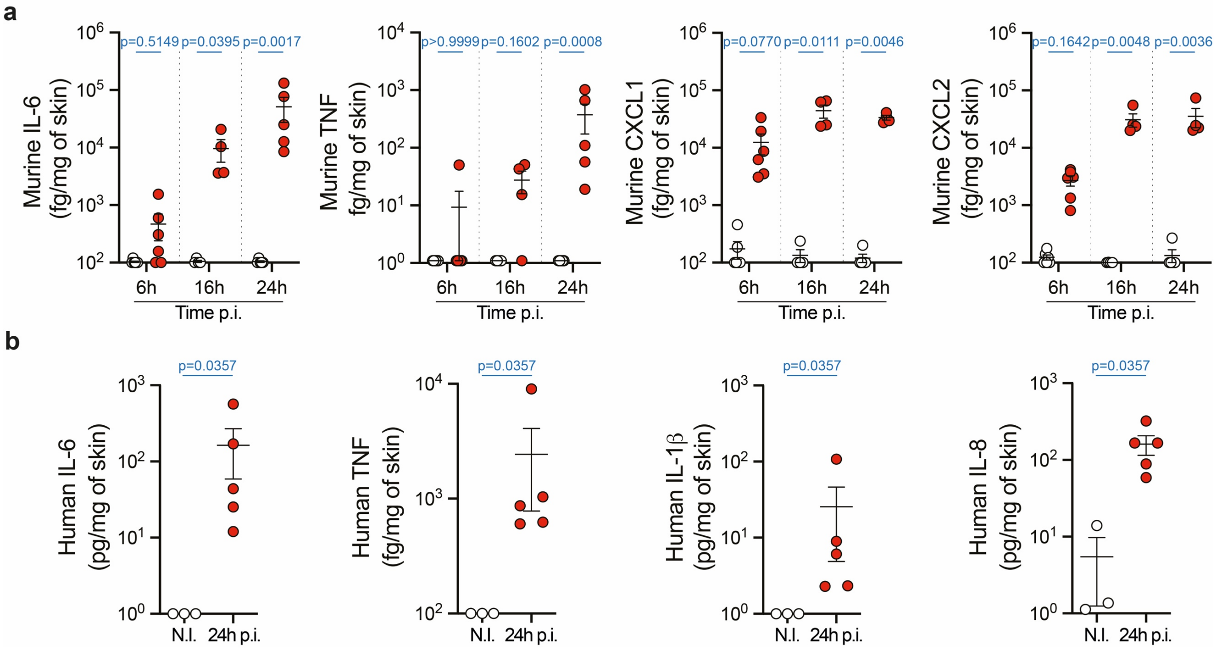

Supplementary Figure 2. Secretion of neutrophil-attracting chemokines by both the murine tissue and the human endothelium upon *Nm* infection. a, Measurements of murine IL-6, TNFα, CXCL1, and CXCL2 levels in whole dissociated xenografts harvested from non-infected (white circles) and infected (red circles) mice at the indicated time points post-infection (p.i.). Two-tailed Kruskal-Wallis test with Dunn's correction. n=6 mice for 6h p.i., 4 mice for 16h p.i. and 5 mice for 24h p.i. per group, pooled from N=2 independent experiments per time point. ns, not significant. **b,** Measurements of human IL-6, TNFα, IL-1β, and IL-8 levels in whole dissociated xenografts harvested from non-infected (white circles) and mice infected for 24h (red circles). Two-tailed Mann-Whitney test. n=3 non-infected mice and 5 infected mice, pooled from N=2 independent experiments. **a-b,** Data are shown as the mean±SEM.

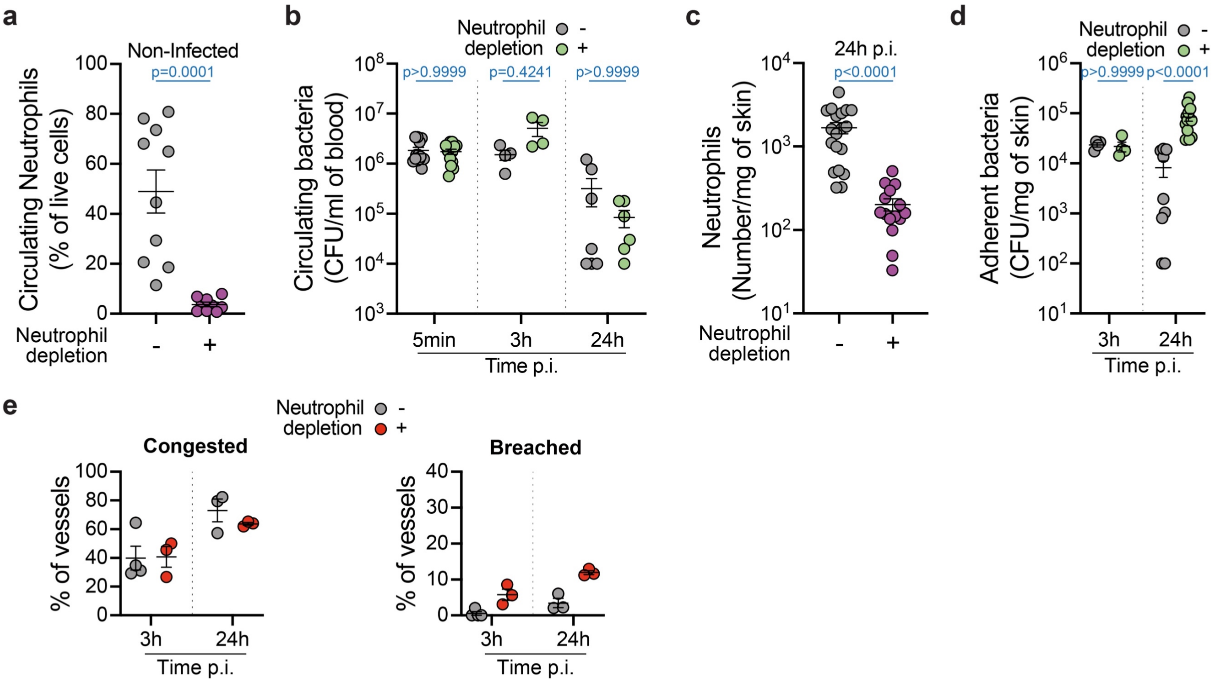

Supplementary Figure 3. Neutrophils limit both vascular colonization by meningococci and vessel damages. a-d, Neutrophil depletion was achieved by intravenous injection of the neutrophil-depleting antibody (anti-Ly-6G, clone 1A8) 24h prior to mouse infection. Mice were sacrificed 24h post-infection and analyses were carried out. **a,** Percentage of blood circulating neutrophils in non-infected mice pre-treated with the isotype control (-, grey circles) or the neutrophil-depleting (+, purple circles) antibody. Two-tailed Unpaired t test. n=10 (control) and 9 (depletion) mice, pooled from N=4 independent experiments. **b,** Bacterial CFU counts from blood of mice pre-treated with the isotype control (-, grey circles) or the neutrophil-depleting (+, green circles) antibody and infected for the indicated times. Two-tailed Kruskal-Wallis test with Dunn's correction. n=13 mice per group (control vs depletion) at 5 min p.i., 4 mice per group at 3h p.i. and 7 and 6 mice for control vs depletion at 24h p.i., respectively, pooled from N=3 independent experiments per time point. ns, not significant. **c,** Neutrophil numbers in xenografts of mice pre-treated with the isotype control (-, grey circles) or the neutrophil-depleting (+, purple circles) antibody and infected for 24h. Two-tailed Man-Whitney test. n=19 (control) and 15 (depletion) mice, pooled from N=5 independent experiments. **d,** Bacterial CFU counts from dissociated xenografts collected from mice pre-treated with the isotype control (-, grey circles) or the neutrophil-depleting (+, green circles) antibody and infected for the indicated times. Two-tailed Kruskal-Wallis test with Dunn's correction. n=4 mice per group at 3h p.i. and 9 (control) and 13 (depletion) mice at 24h p.i., pooled from N=2 (3h p.i.) and 3 (24h p.i.) independent experiments. ns, not significant. **e,** Quantification of vascular damage upon mouse infection for the indicated time points following neutrophil depletion by intravenous injection of the neutrophil-specific depletion antibody (anti-Ly-6G, clone 1A8, red circles) or the isotype control (grey circles) 24h prior to mouse infection. Quantifications were performed on n=200 vessels, pooled from N=3 mice per time point. **a-e,** Data are shown as the mean±SEM.

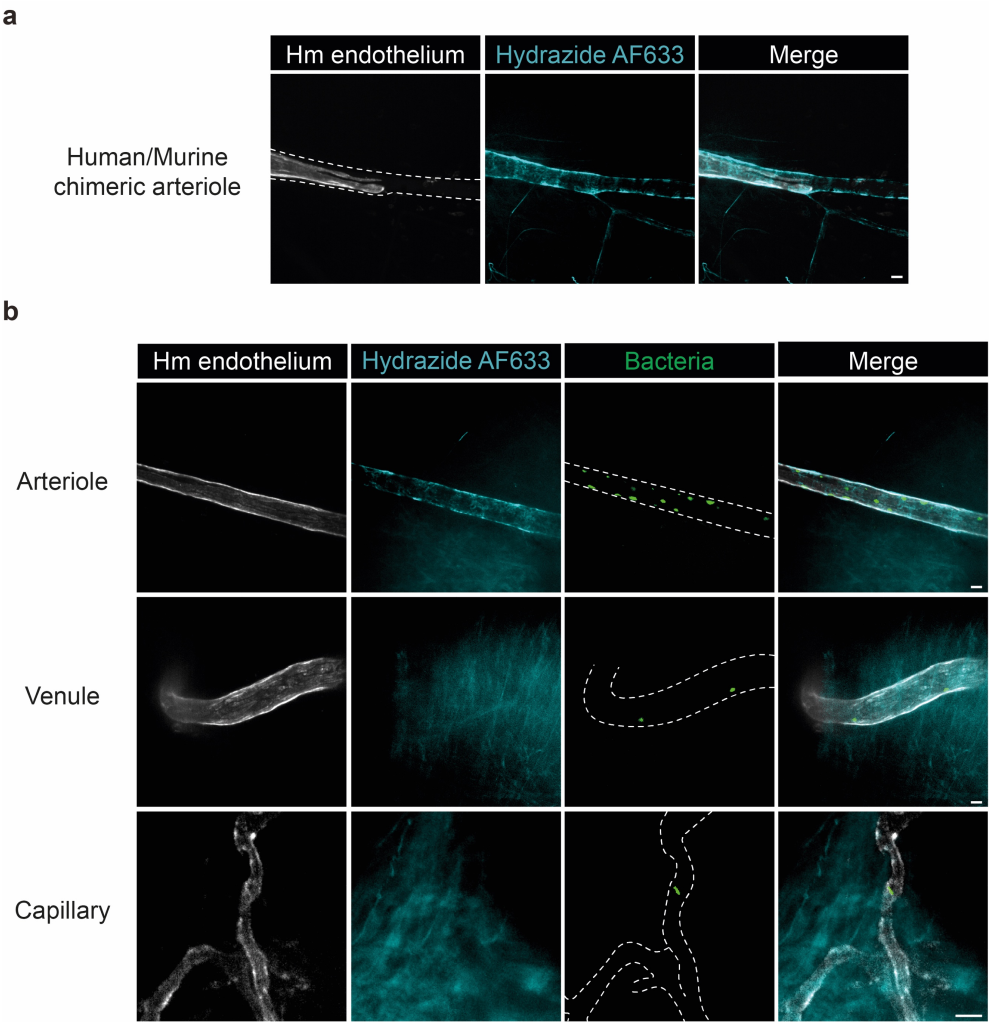

Supplementary Figure 4. Identification of the different vessel types based on hydrazide AlexaFluor633 labelling upon infection. a, Representative images (maximal intensity z-projection) of vessel labelling using Hydrazide AlexaFluor633 (AF633) in a chimeric human/murine arteriole, confirming that Hydrazide AF633 equally stains human and murine arterioles. **b,** Representative images of vascular colonization of the different human vascular types 1h post-infection by GFP-expressing *Neisseria meningitidis* (green) as revealed by the UEA-1 lectin (human endothelium, grey) and Hydrazide AF633 (arterioles, cyan) double labelling. Scale bar, 20 µm. **a-b,** Pictures shown are representative of N=3 mice imaged independently.

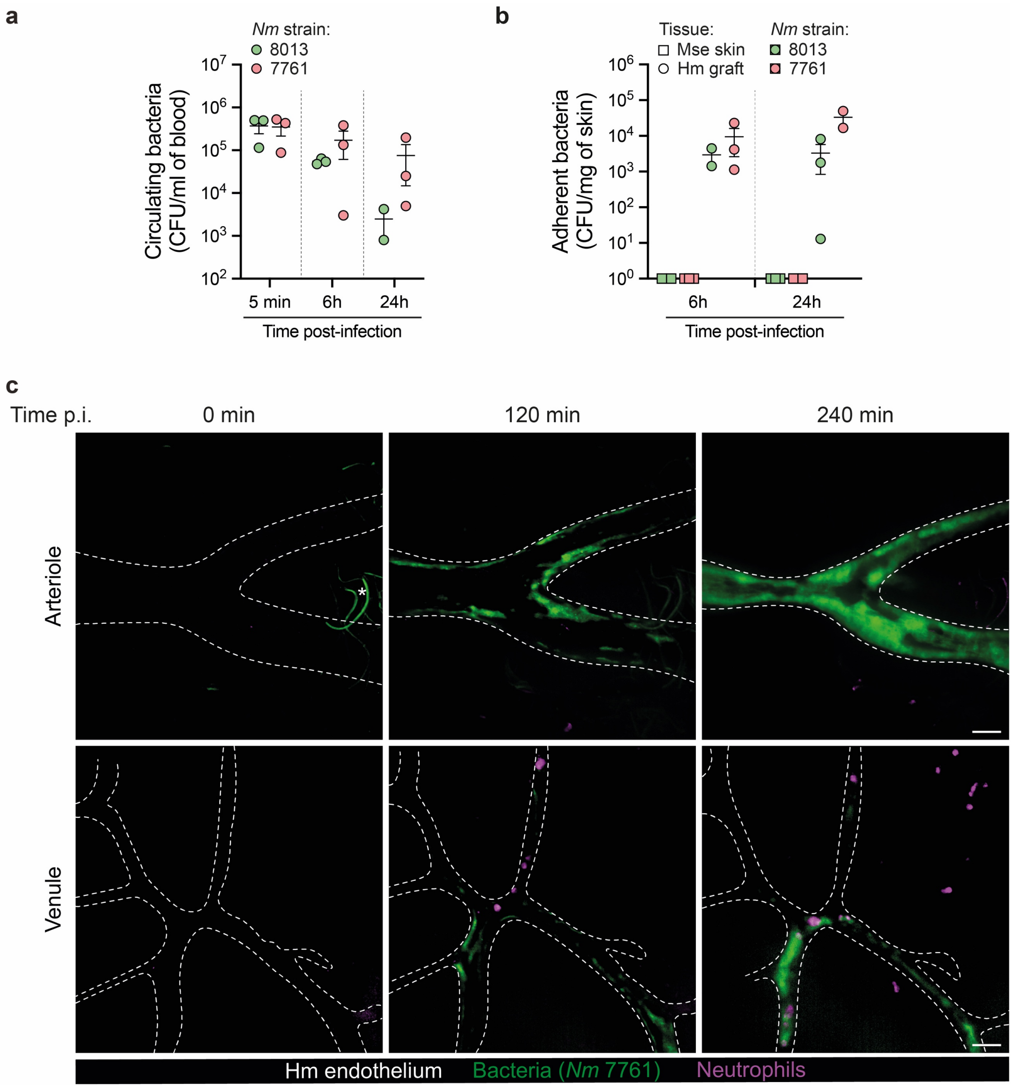

Supplementary Figure 5. The neutrophil response to *Nm* intravascular infection is independent of the bacterial serogroup. a, Bacterial CFU counts from blood of mice infected for the indicated times with meningococcal strains belonging to the serogroup C (strain 8013, green circles) or the serogroup B (strain 7761, light red circles). n=3 mice per group and time points (except for strain 8013, 24h p.i. n=2 mice), pooled from N=2 independent experiments. **b,** Bacterial CFU counts from dissociated mouse skin (squares) or xenografts (circles) collected from mice infected for the indicated times with meningococcal strains belonging to the serogroup C (strain 8013, green) or the serogroup B (strain 7761, light red). n=2 (strain 8013, 6h p.i. and strain 7761, 24h p.i.) and 3 (strain 8013, 24h p.i. and strain 7761, 6h p.i.) mice, pooled from N=2 independent experiments. **a-b,** Data are shown as the mean±SEM. **c,** Representative time sequence (maximal intensity z-projection) of arteriole (top) and venule (bottom) colonization by the meningococcal strain 7761 (serogroup B, green) and highlighting the preferential recruitment of neutrophils (Ly-6G, magenta) to the infected venule. Dashed lines delineate the human endothelium, stained using the UEA-1 lectin. *, tissue autofluorescence. Scale bar, 50 µm.
