# Supplementary material for: Colonization of dermal arterioles by Neisseria meningitidis provides a safe haven from neutrophils
Source: Nat Commun. 2021 Jul 27;12:4547. doi: 10.1038/s41467-021-24797-z (PMC8316345; doi:10.1038/s41467-021-24797-z)
Supplement: Supplementary file 3 — Description of Additional Supplementary Files [file 41467_2021_24797_MOESM3_ESM.pdf]

Description of Additional Supplementary Files

File Name: Supplementary Movie 1
Description: **Vascular colonization by *Neisseria meningitidis.*** Intravital imaging (maximum intensity z-projection) of iRFP-expressing *Neisseria meningitidis* vascular colonization. Bacteria rapidly bind to the human endothelium (UEA-1 lectin, grey) and locally proliferate. At 6 hours post-infection, the 3D-rendering shows the complete colonization of the human endothelium whereas no bacteria were detected within adjacent mouse vessels (mouse CD31, red). Time, hh:min:sec. Scale bar, 50 µm.

File Name: Supplementary Movie 2
Description: **Heterotypic mouse-human interactions.** Intravital visualization (maximum intensity z-projection) of the interaction between mouse neutrophils (Ly-6G, magenta) and TNF[illegible]-mediated inflamed human (UEA-1 lectin, grey) and/or mouse (mouse CD31, red) endothelia. A chimeric human/mouse vessel is shown in the first field of view. Time, hh:min:sec. Scale bar, 20 µm. A segment of a human vessel is shown in the second field of view. Time, hh:min:sec. Scale bar, 10 µm.

File Name: Supplementary Movie 3
Description: **Neutrophil recruitment during the late phase of *Neisseria meningitidis* infection.** Intravital imaging (maximum intensity z-projection) of neutrophil (LysM$^{gfp/+}$, magenta) recruitment 16h post-infection with iRFP-expressing *Nm* (green). Neutrophils were massively recruited around the infected human vessel. Tracking of individual neutrophils showed their directed migration towards the infected human vessel. Time, hh:min:sec. Scale bar, 50 µm.

File Name: Supplementary Movie 4
Description: **Crawling of neutrophils on the human venular endothelium and phagocytosis of adherent *Neisseria meningitidis*.** Intravital visualization (maximum intensity z-projection) of neutrophils (Ly-6G, magenta) internalizing adherent iRFP-expressing *Neisseria meningitidis* (green) inside infected human venules (UEA-1 lectin, grey). In the first field of view, imaging was performed at 3h post-infection. Time, hh:min:sec. Scale bar, 20 µm.

File Name: Supplementary Movie 5
Description: **Perivascular neutrophil recruitment during the early phase of arteriolar colonization by *Neisseria meningitidis.*** Intravital visualization (maximum intensity z-projection) of neutrophil (Ly-6G, magenta) dynamics in the perivascular area of an infected human arteriole (UEA-1 lectin, grey) during the first 6h of infection. Neutrophils remain outside of vessels and do not internalize iRFP-expressing bacteria (green).

File Name: Supplementary Movie 6
Description: **Reduced neutrophil dynamics following vascular colonization of human arterioles.** Intravital visualization (maximum intensity z-projection) of neutrophil (Ly-6G, magenta) dynamics in an infected human arteriole (UEA-1 lectin, grey) 7h post-infection. Neutrophils containing engulfed iRFP-expressing bacteria (green) display a very low motility compared to neutrophils present in the adjacent non-infected mouse venules (mouse CD31, red). Time, hh:min:sec. Scale bar, 20 µm.
