# Supplementary material for: Colonization of dermal arterioles by Neisseria meningitidis provides a safe haven from neutrophils
Source: Nat Commun. 2021 Jul 27;12:4547. doi: 10.1038/s41467-021-24797-z (PMC8316345; doi:10.1038/s41467-021-24797-z)
Supplement: Supplementary file 10 — Reporting Summary [file 41467_2021_24797_MOESM10_ESM.pdf]

nature research

Corresponding author(s): Guillaume Duménil & Dorian Obino

Last updated by author(s): 2021/06/15

Reporting Summary

Nature Research wishes to improve the reproducibility of the work that we publish. This form provides structure for consistency and transparency in reporting. For further information on Nature Research policies, see our Editorial Policies and the Editorial Policy Checklist.

Statistics

For all statistical analyses, confirm that the following items are present in the figure legend, table legend, main text, or Methods section.

n/a	Confirmed	
☐	☒	The exact sample size (n) for each experimental group/condition, given as a discrete number and unit of measurement
☐	☒	A statement on whether measurements were taken from distinct samples or whether the same sample was measured repeatedly
☐	☒	The statistical test(s) used AND whether they are one- or two-sided<br>*Only common tests should be described solely by name; describe more complex techniques in the Methods section.*
☒	☐	A description of all covariates tested
☐	☒	A description of any assumptions or corrections, such as tests of normality and adjustment for multiple comparisons
☐	☒	A full description of the statistical parameters including central tendency (e.g. means) or other basic estimates (e.g. regression coefficient) AND variation (e.g. standard deviation) or associated estimates of uncertainty (e.g. confidence intervals)
☐	☒	For null hypothesis testing, the test statistic (e.g. F, t, r) with confidence intervals, effect sizes, degrees of freedom and P value noted<br>*Give P values as exact values whenever suitable.*
☒	☐	For Bayesian analysis, information on the choice of priors and Markov chain Monte Carlo settings
☒	☐	For hierarchical and complex designs, identification of the appropriate level for tests and full reporting of outcomes
☒	☐	Estimates of effect sizes (e.g. Cohen's d, Pearson's r), indicating how they were calculated

Our web collection on statistics for biologists contains articles on many of the points above.

Software and code

Policy information about availability of computer code

Data collection	BD FACSDiva V7 (BD Bioscience), CytExpert v2.4.0.28 (Beckman Coulter)
Data analysis	FlowJo v10 (Tree Star), Metamorph Imaging Software v7.10.4.407 (Molecular Devices)

For manuscripts utilizing custom algorithms or software that are central to the research but not yet described in published literature, software must be made available to editors and reviewers. We strongly encourage code deposition in a community repository (e.g. GitHub). See the Nature Research guidelines for submitting code & software for further information.

Data

Policy information about availability of data

All manuscripts must include a data availability statement. This statement should provide the following information, where applicable:

- Accession codes, unique identifiers, or web links for publicly available datasets
- A list of figures that have associated raw data
- A description of any restrictions on data availability

Data supporting this work are available in the main figures and the Supplementary Information files. Source data and statistics that underlie the graphs in figures and Supplementary Information are provided with the paper.

Field-specific reporting

Life sciences study design

All studies must disclose on these points even when the disclosure is negative.

Sample size	Sample sizes were not pre-determineted but based on previous literature in the same field of research. The number of mice per group used in the experiments is standard for mouse experiments.
Data exclusions	No data were excluded from the analyses
Replication	All experiments were successfully reproduced by different experimentatators: Fig1a: N=6 mice; Fig.1b: N=3 mice per condition; Fig.1c: N=3 (3h, 6h and 16h p.i.) and 5 (24h p.i.) independent experiments; Fig.1d: N=7 infected mice imaged independently; Fig.1e: N=3 mice imaged independently; Fig.2b-d: N=3 independent experiments (except for pilD N=1); Fig.2f-g: N=3 independent experiments; Fig.2h: N=2 independent experiments; Fig.3b-d: N=4 independent experiments; Fig.3e: N=3 independent experiments; Fig.3f: N=5 (Non-infected), 4 (3h p.i.) and 3 (6h, 16h and 24h p.i.) mice; Fig.3g: N=3 mice per group; Fig.3h: N=4 independent experiments; Fig.4a: N=3 independent experiments; Fig.4b: N=3 mice per condition; Fig.4c: N=3 independent experiments; Fig.4d-e and g-h: N=7 infected mice imaged independently; Fig.5a-c and Fig.6a-c: N=7 infected mice imaged independently. Fig.7a: N=3 independent experiments; Fig.7b-c: N=3 infected mice imaged independently; Fig.7d: N=3 infected mice imaged independently per condition; Fig.7e: N=3 infected mice imaged independently. Only the pictures shown in Fig. 4f correspond to one single human donor.
Randomization	Mice (control and grafted) were randomly assigned to the different conditions tested. For Flow cytometry analyses on cultured cells, cells from the same cultures were divided into the different conditions. Each treatment was randomly applied to the cell sub-cultures.
Blinding	Investigators were not blinded during data collection since data collection was performed by the same individual who performed the mouse infections. Data analyses were performed semi-bindly: 1 no-blinded investigator (the one who infected mice and collected the data) and another blinded investigator (different from the one who collected the data) analyzing he data in parallel.

Reporting for specific materials, systems and methods

We require information from authors about some types of materials, experimental systems and methods used in many studies. Here, indicate whether each material, system or method listed is relevant to your study. If you are not sure if a list item applies to your research, read the appropriate section before selecting a response.

Materials & experimental systems

n/a	Involved in the study	
☐	☒	Antibodies
☐	☒	Eukaryotic cell lines
☒	☐	Palaeontology and archaeology
☐	☒	Animals and other organisms
☐	☒	Human research participants
☒	☐	Clinical data
☒	☐	Dual use research of concern

Methods

n/a	Involved in the study	
☒	☐	ChIP-seq
☐	☒	Flow cytometry
☒	☐	MRI-based neuroimaging

Antibodies

Antibodies used

The following antibodies have been used in this study:
For flow cytometry:
From Biolegend: PacificBlue-conjugated anti-Ly-6C (clone HK1.4, #128014), PacificBlue- or PE/Cy7-conjugated anti-Ly-6G/Ly-6C (GR-1) (clone RB6-8C5, #108430 or #108416), PacificBlue-conjugated anti-CD45 (clone 30-F11, #103126).
From eBioscience: APC-conjugated anti-CD11b (clone M1/70, #17-0112-82), PE-conjugated anti-Ly-6C (clone HK1.4, #12-5932-82), PE-Cy7-conjugated anti-Human CD106/VCAM-1 (clone STA, #25-1069-42), PE-conjugated anti-Human CD62E/E-Selectin (clone P2H3, #12-0627-42).
From BD Biosciences: BUV395-conjugated anti-CD45 (clone 30-F11, #564279), rat anti-mouse CD16/CD32 (FcBlock clone 2.4G2, #553141).
From Life Technologies: AlexaFluor700-conjugated anti-Human CD54/ICAM-1 (clone 1H4, #MA528553).
For immunostaining of tissue sections:
Rat anti-mouse Ly-6C/Ly-6G (RB6-8C5, BD Bioscience, #550291) followed by AlexaFluor647-conjugated Goat anti-Rat IgG (ThermoFisher Scientific, #A21247).
For intravital microscopy:
Rat anti-mouse CD31 (clone 390, Biolegend, #102412), Rat anti-mouse Ly-6G (clone 1A8, Biolegend, #127620), PE-conjugated anti-human CD62E (clone P2H3, Thermofisher Scientific, #12-0627-42), anti-E-Selectin blocking antibody (clone P2H3, Thermofisher Scientific, #14-0627-82), mouse IgG1kappa isotype control (clone P3.6.2.8.1, Thermofisher Scientific, #14-4714-85).
For in vivo neutrophil depletion:
Rat anti-mouse Ly-6G/Ly-6C (GR-1) (clone RB6-8C5, eBioscience, #16-5931-85),Rat anti-mouse Ly-6G (clone 1A8, eBioscience, #16-9668-85), Rat IgG2beta,kappa or eBrD isotype controls (eBioscience, #16-4031-85 and #14-4321-85, respectively).

Validation	Validation of all primary antibodies for the species and application was warranty by the suppliers. Validation statements can be found on the manufacturer's website.

Eukaryotic cell lines

Policy information about cell lines

Cell line source(s)	HUVECs: LONZA
Authentication	Authentification performed by the supplier using specific cell surface markers
Mycoplasma contamination	Checked by the supplier - All cultures were negative for mycoplasma contamination
Commonly misidentified lines (See ICLAC register)	No commonly misidentified lines were used in this study

Animals and other organisms

Policy information about studies involving animals; ARRIVE guidelines recommended for reporting animal research

Laboratory animals	Mice, Strains: SCID/Beige (CB17.Cg-PrkdcscidLystbg-J/Crl), C57BL/6 Rag2-/- γc-/- LysMgfp/+. For all experiments, male and female mice between 6 and 10-weeks of age were used
Wild animals	This study did not involved wild animals
Field-collected samples	This study did not involved samples collected from the field
Ethics oversight	All experiments were performed in agreement with guidelines established by the French and European regulations for the care and use of laboratory animals and approved by the Institut Pasteur committee on Animal Welfare (CETEA) under the protocol code CETEA 2015–0025.<br>Human skin samples (xenograft mouse model) were used in accordance with the French legislation, patients were informed and did not refuse to participate in the study. All procedures were approved by the local ethical committee Comité d'Evaluation Ethique de l'INSERM IRB 00003888 FWA 00005881, Paris, France Opinion: 11-048.

Note that full information on the approval of the study protocol must also be provided in the manuscript.

Human research participants

Policy information about studies involving human research participants

Population characteristics	The experimentators were blinded regarding sex and age of the human skin donors. Skin biopsies from men and women were used without any specific inclusion/exclusion criteria (age, sex, orgin).
Recruitment	Donors were recruited and informed by Dr. Taliah Schmitt. They did not refuse to participate in the study and signed a consent form to give their skin surgerical waste to research, in accordance with the French legislation
Ethics oversight	All procedures were approved by the local ethical committee Comité d'Evaluation Ethique de l'INSERM IRB 00003888 FWA 00005881, Paris, France Opinion: 11-048.

Note that full information on the approval of the study protocol must also be provided in the manuscript.

Flow Cytometry

Plots

Confirm that:

- [x] The axis labels state the marker and fluorochrome used (e.g. CD4-FITC).
- [x] The axis scales are clearly visible. Include numbers along axes only for bottom left plot of group (a 'group' is an analysis of identical markers).
- [x] All plots are contour plots with outliers or pseudocolor plots.
- [x] A numerical value for number of cells or percentage (with statistics) is provided.

Methodology

Sample preparation	From skin biopsies: human skin xenograft and/or mouse skin biopsies were collected immediately after mouse sacrifice and weighed on a fine balance. Biopsies were cut into small pieces and digested with gentle agitation for 60-90 min at 37°C in CO2-independent medium containing 25 mM Hepes, 0.4 mg/ml Liberase TL (Sigma Aldrich), 0.04 mg/ml DNase I (Sigma Aldrich), and 100 U/ml Penicillin/Streptomycin (Gibco). The resulting single cell-suspension was passed through a 70-µm cell strainer (BD Bioscience) and treated with 1x RBC lysis solution (BioLegend). Cells were again filter using 40-µm cell strainer (BD Bioscience) and counted. Cell viability was determined using trypan blue exclusion. Single-cell suspensions were then aliquoted by 1.10E6 cells per 100 µl in 1x PBS supplemented with 2% FBS. Fc receptors were blocked using the anti-mouse

	CD16/CD32 (FcBlock clone 2.4G2) monoclonal antibodies (BD Biosciences, #553141, 1/400) and cells were stained for 30 min at 4°C with a combination of anti mouse antibodies. Exclusion of non-viable cells has been achieved using eFluor780 Fixable viability dye (eBioscience, #65-0865) according to manufacturer's instructions. After staining, cells were washed in 1x PBS and fixed for 20 min at 4°C with 4% paraformaldehyde in 1x PBS and washed with 1x PBS supplemented with 2% FBS.<br>From cultured endothelial cells: Following treatment, cells were harvested using 37°C preheated Versene (Gibco, #15040066) to preserve cell surface epitopes. Fc receptors were blocked using Human TruStain FcX (BioLegend, #422302, 1/20) diluted in cold FACS buffer (1x PBS supplemented with 0.5% BSA and 2 mM EDTA). Cells were stained for 30 min on ice with a combination of anti-human antibodies diluted in cold FACS buffer. Exclusion of non-viable cells has been achieved using eFluor506 Fixable viability dye (eBioscience, # 65-0866-14) according to manufacturer's instructions. After staining, cells were washed in cold 1x PBS and fixed over-night at 4°C with 1% paraformaldehyde in 1x PBS.
Instrument	BD LSR Fortessa & CytoFLEX LX from Beckman Coulter
Software	Data collection using the LSR Fortessa: BD FACSDiva v7 software<br>Data collection using the CytoFLEX LX: CytExpert v2.4.0.28 Software<br>Data analysis: FlowJo v10 software (Tree Star, Ashland, OR, USA)
Cell population abundance	The cell population abundance is the parameter under study in this paper: we assessed the local recruitment of neutrophils upon intravascular infection. From the literature on pathogen-induced neutrophil recruitment within tissues, we expected few neutrophils in controls conditions, with numbers/proportions increasing upon infections.
Gating strategy	Gating strategy to quantify neutrophil numbers is provided in Supplementary Figure 1C and is based on the sequential gating of the 'cells' among 'all events'. Then, using FSC-W and SSC-W parameters, 'singlets' were selected. 'Singlets' negative for the Viability dye were selected as 'Live singlet cells'. 'Myeloid cells' were selected from the 'Live singlet cells' based on the co-expression of CD45 and CD11b. Finally, 'neutrophils' were selected from the 'Myeloid cells' based on the co-expression of the neutrophil-specific markers GR-1 and Ly-6C.

☒ Tick this box to confirm that a figure exemplifying the gating strategy is provided in the Supplementary Information.
