# Supplementary material for: Colonization of dermal arterioles by Neisseria meningitidis provides a safe haven from neutrophils
Source: Nat Commun. 2021 Jul 27;12:4547. doi: 10.1038/s41467-021-24797-z (PMC8316345; doi:10.1038/s41467-021-24797-z)
Supplement: Supplementary file 2 — Peer Review File [file 41467_2021_24797_MOESM2_ESM.pdf]

Editorial Note: This manuscript has been previously reviewed at another journal that is not operating a transparent peer review scheme. This document only contains reviewer comments and rebuttal letters for versions considered at *Nature Communications*.

REVIEWERS' COMMENTS

Reviewer #1 (Remarks to the Author):

The authors have done a nice job of answering the previous reviews by adding references, text, and new data. This is an important addition to the literature and clearly moves this field forward.
Hank Seifert

Reviewer #2 (Remarks to the Author):

Accept

Reviewer #3 (Remarks to the Author):

The authors have addressed all my previous points and added novel mechanistic data to the study. I recommend it for publication.

REVIEWERS' COMMENTS

Reviewer #1 (Remarks to the Author):

The authors have done a nice job of answering the previous reviews by adding references, text, and new data. This is an important addition to the literature and clearly moves this field forward.
Hank Seifert

Reviewer #2 (Remarks to the Author):

Accept

Reviewer #3 (Remarks to the Author):

The authors have addressed all my previous points and added novel mechanistic data to the study. I recommend it for publication.

RESPONSE TO REVIEWERS' COMMENTS

We are glad the reviewers found our work an important contribution to the field and thank them for their supportive comments.
